# EHMTI-0371. A new physical sign in migraine 'pointing forehead'

**DOI:** 10.1186/1129-2377-15-S1-J11

**Published:** 2014-09-18

**Authors:** S Samarakoon, A Munasinghe

**Affiliations:** 1 Health, Ministry of Health, Colombo, Sri Lanka

## Objectives

This study was designed to compare the presence of seven clinical signs in a group of patients with migraine with that of patients with non-migraine headache.

## Background

Migraine is sometimes misdiagnosed. Therefore additional features are useful to improve the diagnostic accuracy of migraine.

## Methods

A cross sectional descriptive study was conducted in a group of 709 outpatients with headache. The physical signs were named as A-G. These were carefully observed certain gestures exhibited by patients themselves when they describe their headache.

## Results

Sign A (pointing right side of the forehead) and sign B (pointing left side of the forehead) were significantly higher in patients with migraine (Sign A positive-123/339, Chi square-15.784, p<0.001; Sign B positive-146/339, Chi square-20.813, p<0.001). Sign F (keeping the head on a table) was significantly higher in patients with non-migraine headache (Sign F positive-132/370, Chi square-12.954, p<0.001). Sign A was more commonly associated with unilateral, severe headache which lasted for a longer period of time. However sign B was more commonly associated with unilateral, severe headache only. Sign C was significant in patients who had bilateral headache in both migraine and non-migraine groups than unilateral headache.

## Conclusions

It is concluded that pointing right or left side of forehead when the patient describes his or her headache is a characteristic sign of migraine. Keeping the head on the table during an attack of headache is not a characteristic sign of migraine.

